# Gender differences in marriage, romantic involvement, and desire for romantic involvement among older African Americans

**DOI:** 10.1371/journal.pone.0233836

**Published:** 2020-05-29

**Authors:** Dawne M. Mouzon, Robert Joseph Taylor, Linda M. Chatters

**Affiliations:** 1 Edward J. Bloustein School of Planning and Public Policy, Rutgers, The State University of New Jersey, New Brunswick, New Jersey, United States of America; 2 School of Social Work, University of Michigan, Ann Arbor, Michigan, United States of America; 3 Institute for Social Research, Program for Research on Black Americans, University of Michigan, Ann Arbor, Michigan, United States of America; 4 School of Public Health, University of Michigan, Ann Arbor, Michigan, United States of America; Indiana University Purdue University at Indianapolis, UNITED STATES

## Abstract

**Background:**

Despite research on the dramatic changes in marriage, there is a dearth of research on the correlates of marriage and romantic involvement among older African Americans. This is an important omission because although the marriage decline is universal, African Americans show the steepest decline in marriage rates.

**Methods:**

Based on data from the National Survey of American Life, multinomial logistic regression analysis is used to identify demographic and health correlates of: 1) being married or cohabiting, 2) having a romantic involvement, 3) not having a romantic involvement but desiring one, and lastly, 4) not having and not desiring a romantic involvement.

**Results:**

Four in 10 older African Americans are either married or cohabiting, 11% are unmarried but romantically involved, 9.5% are unmarried and not romantically involved but open to the possibility of a relationship, and 38% neither have nor desire a romantic involvement. More men than women are married or cohabiting, a gap that increases with advanced age. Across all age groups, African American women are more likely than their male counterparts to report that they neither have nor desire a romantic relationship.

**Conclusion:**

Almost as many older African Americans do not want a romantic relationship as those who are married/cohabiting. Findings support social exchange theories and the importance of an unbalanced sex ratio. Furthermore, the results suggest that singlehood among older African Americans (especially women) is not necessarily an involuntary status. Nonetheless, this group is at higher risk of economic and health problems as they age.

## Introduction

The drastic decline in U.S. marriage rates over the past 50 years is one of the most striking demographic trends in recent history. In the United States, approximately 68% of men and 66% of women were married in 1950, compared to only 54% of men and 51% of women in 2019 [1]. Marriage decline, along with older age at first marriage [2], growth of non-marital cohabitation [3], and increases in remarriage rates [4], reflect important demographic trends in marriage and relationship behaviors. Significant demographic changes also impact older adults, a population that is expected to double between 2012 and 2050 [5]. Although divorce rates have generally remained stable since the 1960s, the divorce rate among adults 50 years and older actually doubled between 1990–2010, a trend dubbed the “gray divorce revolution” [6]. Roughly one-third of Baby Boomers (born between 1946–1964) are currently unmarried [7].

Men’s shorter life expectancy [8], coupled with women’s lower propensity to remarry [4], creates a gender gap in the prevalence of marriage among older adults. For example, after age 65, 71% of men but only 42% of women are currently married, indicating that a growing population of older adults–especially women—are likely to live outside of the context of marriage [9]. Additionally, African Americans have the lowest marriage rates [2] and largest gender gap in marriage among all race/ethnic groups [10]. Therefore, findings from marriage and relationship research (using predominantly White samples) cannot be generalized to African Americans. We seek to remedy this gap by investigating the sociodemographic and health correlates of marriage, romantic involvement, and desire for a romantic union among a national sample of older African Americans.

A major emphasis of this study is also examining non-marital romantic relationships among older African Americans. Beginning in the early 2000s, research in the United States began documenting the rise of adult non-marital romantic relationships using the northern European phrase “Living Apart Together” or LAT. This is evident in the title of Levin’s article (2004) “Living apart together: A new family form” [11]. However, it is important to note that in the early 1990s, Tucker and colleagues [12] examined this issue among older African Americans. Tucker and colleagues did not use the term LAT but instead used the term “main romantic involvement.” This was preferred to other terms that African American adults deemed inappropriate for their age and status (i.e., boyfriend, girlfriend, going steady).

## Literature review

Theoretical perspectives on relationship formation and marriage identify several factors that potential partners consider before entering romantic unions. The present analysis is framed by two related perspectives on marital and relationship behaviors—*social exchange theory* (assessments of costs and benefits of unions) and the *sex ratio imbalance* (disparities in the proportion of females and males). These perspectives operate in a complementary manner in characterizing the broader context within which opportunities, constraints, and decisions about marriage and relationship formation for African Americans occur. As discussed below, social exchange theory’s propositions regarding relationship formation and marriage patterns and the specific demographic (e.g., sex ratio imbalance) and social contexts of African Americans are mutually constitutive of their unique dating and marriage markets.

### Social exchange theory and marriage and relationship formation

Social exchange theories [13,14] emphasize individuals’ desire to enter into social relationships (including romantic relationships) in which they can maximize their gains and minimize their costs of investment. Individuals employ a rational choice approach in deciding whether to enter a romantic relationship by weighing both economic and non-economic advantages and disadvantages. If the costs of investing in a social relationship exceed the expected benefits, individuals often decide not to engage. If a satisfactory balance of costs and rewards (e.g., perceived reciprocity) is maintained, the social relationship continues. If the cost-reward balance shifts over the course of a social relationship, individuals may choose to disengage. Perceived alternatives to the current partner is another factor influencing decisions to initiate and maintain relationships [15]. Individuals may decide to invest in an alternative relationship given a perceived opportunity to attain a more favorable cost-reward balance (e.g., potential partners with valued resources and/or traits). Applied to marriage and romantic involvement, social exchange theory can be used to understand how demographic (e.g., age, gender, income) and health factors operate in relation to decisions to engage, sustain, or leave intimate partnerships.

Consistent with social exchange theory, prior research indicates that sociodemographic characteristics are important correlates of marriage and romantic involvement. Marriage is strongly associated with socioeconomic status; persons of higher SES are more likely to become and remain married [2,16,17]. Age is also inversely associated with dating, marriage, and the desire for marriage and remarriage [2,18–20]. Good health status increases the likelihood of entering into (and remaining in) an intimate partnership [21,22]. Although for men, ‘adverse selection’ may occur whereby men in poor health (who view marriage as a means of attaining health benefits) are more likely to remarry and remain married longer [23].

### Sex ratio imbalance and marriage and relationship formation among older African Americans

Overall, Black Americans have experienced a dramatic decrease in marriage rates over time, from 64% of Black men and 62% of Black women being married in 1950 to 38% of Black men and 33% of Black women being married in 2019 [1]. Black Americans also have higher divorce rates and lower remarriage rates than Whites [4,24]. Gender and age patterns for marriage and relationship status indicate that older Black women, in particular, are less likely to be married than women in other race/gender groups [9]. These disparate marriage patterns are associated with the disproportionate sex ratio (i.e., alive and noninstitutionalized potential partners) for Blacks, whereby there are roughly 79.0 Black men for every 100 Black women in 2019, compared to 91.8 White men for every 100 White women in 2019 [1].

The sex ratio imbalance itself is associated with two main factors impacting Black men—reduced health status and life expectancy and mass incarceration. First, Black men have a life expectancy of only 71.5 years, which is shorter than any other race/ethnic group. Black men live 6.6 fewer years than Black women, 4.6 fewer years than White men, and 9.5 fewer years than White women [25]. Second, as a consequence of mass incarceration policies, Black men are incarcerated at a rate that is six times higher than that of White men [26], resulting in their systematic physical removal from dating and marriage markets. Viewed over the life course, this results in their exclusion from dating and marriage markets during early and middle adulthood, which are typical ages for relationship formation. These two factors are important in shaping marriage and dating contexts and have implications for how social exchange theories operate in relation to African American marriage and relationship behaviors.

Poor health profiles and decreased life expectancy, coupled with high rates of incarceration, reduces the availability of Black men as potential marriage partners. According to social exchange theory, the resulting sex ratio imbalance shifts the advantage in finding a partner to Black men rather than Black women. However, for those men who have experienced incarceration, its long-term effects (including reduced job and employment prospects, lower earned income, and housing restrictions) [27] severely limit their attractiveness as dating and marriage prospects. Further, from the perspective of social exchange theory, Black men in the community who are available as potential partners still face myriad socioeconomic inequities that often make them less “marriageable” [28]. For example, women strongly prefer to date and marry men with stable employment and high levels of education and income [29]—a preference that is especially prominent among Black women [30]. However, studies of socioeconomic attainment indicate that Black men still experience higher levels of unemployment and lower median income than White men with comparable levels of education and are less likely to benefit from investments in education [31].

With respect to the marriage context for older African Americans, Tucker and colleagues [12] examined demographic (age, gender, education, and income), economic, and structural perspectives on marital and relationships behaviors among persons 55 years and older from the nationally representative National Survey of Black Americans (1979–1980). Their work indicated that, for older African Americans in particular, relationship status and formation occur within a distinctive context of a skewed female-male gender ratio that affects dating and marriage markets [12,32]. Further, the marriage decline continued to accelerate after the years in which these data were collected. The present study seeks to investigate these relationships with more recent data, in light of rapidly changing demographic patterns.

In sum, social exchange theory and related research on the correlates of marital and relationship behaviors must be viewed within the demographic (e.g., sex ratio imbalance), structural, and economic factors that shape the social context of marriage and dating for older African Americans [33,34]. Taken together, this information indicates that research on marriage and relationship status among older African Americans should consider how contemporary demographic and social context factors are associated with marriage and relationship attitudes and outcomes (marriage, cohabitation, romantically involved). Specific demographic (i.e., age and gender) and contextual factors may corroborate prior research findings regarding relationship behaviors of older African Americans, while others may be inconsistently related to outcomes.

## Focus of the current study

The present study builds upon research on intimate relationship status of older African Americans using nationally representative data on African American adults aged 55 and older from the National Survey of American Life (2001–2003). Multinomial logistic regression analyses are used to examine sociodemographic and health factors associated with having a romantic involvement, being unpartnered and desiring a romantic involvement, and being unpartnered and not desiring a romantic involvement. We expect that, similar to previous findings [12] and consistent with social exchange theory, younger age and male gender will be associated with being partnered (married or in a romantic union) as well as desiring a romantic union. For unpartnered respondents who do not desire a romantic union, female gender and older age will be associated with this preference. Further, because this analysis seeks to understand the ways in which both gender and age are associated with marriage, romantic involvement, and desire for a romantic union, we explore interactive effects of age and gender for several relationships.

Previous findings [12] for income and education effects among older African Americans are mixed, with income being positively associated with having a romantic involvement but negatively associated with marriage, while education is negatively associated with a current romantic union but unrelated to marriage. Similar to social exchange theory, we anticipate that higher income and education reflect valued resources that are associated with a higher likelihood of being partnered (married/cohabitating) as compared to other relationship statuses. Our study also examines a measure of material hardship with the expectation that low levels of material hardship will be associated with being married/partnered. Given a more traditional and religious milieu and culture [35,36] that supports marriage, we expect that residence in the South will be associated with higher likelihood of marriage (as compared to non-South regions). Finally, due to shortened life expectancy for African Americans (especially men), health status will be a significant factor for intimate relationship behaviors and intentions such that better health will be associated with marriage or having a romantic relationship.

## Methods

### Sample

The National Survey of American Life: Coping with Stress in the 21st Century (NSAL) was collected by the Program for Research on Black Americans at the University of Michigan’s Institute for Social Research [37]. A total of 6,082 interviews were conducted with adults aged 18 or older, including 3,570 African Americans. In this study, we focus only on the sub-sample of older African Americans aged 55 years or older. After accounting for missing data, the analytic sample for this analysis is 766. The NSAL sample utilized a national multi-stage probability design with an overall response rate of 72.3%. Data collection was conducted from 2001 to 2003 and respondents were compensated for their time; a more detailed discussion of the NSAL sample can be found elsewhere [37]. The NSAL is publicly available for downloading from the website of the social science data sharing archive, Inter-University Consortium of Political and Social Research. It has been downloaded over 1,000 times and can be analyzed online.

### Measures

The main outcome for this investigation was *romantic involvement/desire*, a composite measure based on marital status and two measures of relationship status. Marital status was assessed using a single item that asked respondents if they were currently: married, living with a partner, separated, divorced, widowed, or never married. Analyses revealed that only 14 respondents reported that they were currently cohabiting with a partner (weighted percent = 1.9%). Consequently, they were combined with married respondents into a single category for “married/cohabiting.”

All currently unmarried, non-cohabiting respondents were additionally asked whether they were currently involved in a romantic relationship. Respondents who indicated that they were not involved in a romantic relationship were then asked, “Do you want a main romantic involvement?” The responses for these questions were combined to construct a four-category measure of romantic involvement: 1) currently married or cohabiting, 2) has a romantic relationship, 3) unpartnered but desire a romantic relationship, and 4) unpartnered and does not desire a romantic relationship. This variable measures respondents’ current relationship status and all categories are mutually exclusive. We use the term “romantic involvement” (instead of “living apart together”) to denote unmarried adults who are romantically involved, but who live in separate residences. This decision was based on focus group data showing that Black adults preferred this term; moreover, “romantic involvement” is consistent with previous research on Black Americans [12,32].

Several demographic and health factors were included as control variables. Both *age* and *educational attainment* were measured in number of years. For the total NSAL sample, missing data for *household income* were imputed for 773 cases (12.7% of the total NSAL sample) and missing data for *education* were imputed for 74 cases (1.2% of the total NSAL sample). *Region* was coded as Northeast, North Central, West, or South. *Material hardship* was a summary score of seven items assessing whether or not respondents: could meet basic expenses, could pay full rent or mortgage, could pay full utilities, had utilities disconnected, had telephone disconnected, were evicted for non-payment, or could not afford leisure activities in the past 12 months. Higher scores on this scale indicated higher levels of economic hardship (α = 0.76). The question for *self-rated physical health* asked: “How would you rate your overall physical health at the present time?” using five categories including poor (1), fair (2), good (3), very good (4), and excellent (5).

### Analytic strategy

Seventy-one respondents with missing data on any variables were excluded from analyses. The use of listwise deletion in cases where missing data represents less than 10% of the sample is acceptable, having little impact on the validity of statistical inferences [38]. Descriptive statistics are presented as weighted proportions based on the distribution of African Americans in the population. Bivariate analyses used the Rao-Scott chi-square, a complex design-corrected measure of association. Multinomial logistic regression was used to conduct the multivariate analyses. Multinomial logistic regression is appropriate for the four-level polytomous response outcome variable used in this study (i.e., *romantic involvement/desire*) and can accommodate both continuous and categorical independent variables.

The results focus on five unique comparisons. Three of the comparisons involve individuals who are married or cohabiting: 1) romantically involved vs. married/cohabiting; 2) desire a main romantic involvement vs. married/cohabiting; 3) neither has nor desires a main romantic involvement vs. married/cohabiting. Another two unique comparisons were made for the sample of respondents who neither have nor desire a main romantic involvement: 1) romantically involved vs. neither have nor desire a main romantic involvement; and 2) desire a main romantic involvement vs. neither have nor desire a main romantic involvement.

For the multinomial logistic regression analyses, relative risk ratios (RRR) and 95% confidence intervals are presented. Based upon findings from the bivariate analyses, all multinomial logistic regression analyses include an interaction term between age and gender. The analyses were conducted using SAS 9.13 and Stata 12.1, which uses the Taylor expansion approximation technique for calculating the complex design-based estimates of variance. All statistical analyses utilize analytic weights to obtain results that are generalizable to the African American population. All analyses also account for the complex multistage clustered design of the NSAL sample, unequal probabilities of selection, nonresponse, and post-stratification to calculate weighted, nationally representative population estimates and standard errors.

## Results

Descriptive data for all variables are presented in Table 1. Roughly 59.3% of respondents were women and the mean age of the analytic sample was 66.5 years (SD: 7.30). Average years of educational attainment was 11.5 (SD: 2.95 years), mean household income was $33,537 (SD: $33,767) and more than half (55.9%) of respondents lived in the South. The mean level of material hardship was 0.44 (SD: 0.84, range: 0–7). Respondents generally averaged good self-rated health (mean: 3.08, SD: 0.93). In terms of romantic involvement, 40.7% of respondents were married or cohabiting and 11.3% were romantically involved (with a non-residential partner). Roughly 9.5% of respondents were not currently involved in a romantic relationship but would like to be; conversely, 38.5% of respondents neither had nor desired a main romantic involvement (Table 1).

**Table 1:** Demographic characteristics of the sample and distribution of study variables, African Americans aged 55 and older, National Survey of American Life (N = 766).

	%	N	Mean	S.D.	Min	Max
Gender						
Male	40.68	273				
Female	59.32	493				
Age		766	66.48	7.30	55	93
Education		766	11.53	2.95	0	17
Household Income		766	33537	33767	0	450000
Region						
South	55.86	483				
Northeast	16.54	102				
Midwest	17.97	133				
West	9.64	48				
Material Hardship (#)^a^		766	0.44	0.84	0	7
Self-Rated Health^b^		766	3.08	0.93	1	5
Marital and Romantic Status						
Married and Cohabiting	40.73	237				
Romantically Involved	11.32	98				
Desire Romantic Involvement	9.50	81				
Neither has nor desire main romantic involvement	38.45	350				

^a^ Material hardship ranges from 0 to 7 events.

^b^ Self-rated health ranged from 1/poor to 5/excellent.

Percents and N are presented for categorical variables. Means and standard deviations are presented for continuous variables. Unweighted frequencies and weighted percentages are presented.

Gender was strongly associated with patterns of romantic involvement and desire among older African Americans (Table 2). For example, more than half (57.3%) of older African American men were married or cohabiting, compared to only 29.4% of older African American women. Conversely, almost half of older African American women (49.7%) neither had nor desired a main romantic involvement, compared to only 22.0% of older African American men.

**Table 2:** Gender differences in romantic involvement/desire, African Americans aged 55 and older, National Survey of American Life (N = 766).

	Men (N = 273)		Women (N = 493)		Total (N = 766)
	N	%	N	%	N
**Romantic Involvement/Desire**					
Married or cohabiting	126	57.33	111	29.35	237
Romantically involved	45	13.14	53	10.07	98
Unpartnered but desire romantic involvement	28	7.51	53	10.86	81
Neither has nor desire main romantic involvement	74	22.01	276	49.72	350
***BY AGE GROUP***					
**55–64 Years**					
**Romantic Involvement/Desire**					
Married or cohabiting	59	54.04	73	42.13	132
Romantically involved	25	16.55	33	12.77	58
Unpartnered but desire romantic involvement	20	11.96	34	13.74	54
Neither has nor desire main romantic involvement	27	17.45	82	31.35	109
**TOTAL**	131		222		353
**65–74 Years**					
**Romantic Involvement/Desire**					
Married or cohabiting	43	61.85	32	21.22	75
Romantically involved	15	11.05	16	8.93	31
Unpartnered but desire romantic involvement	7	4.40	17	11.25	24
Neither has nor desire main romantic involvement	31	22.69	116	58.60	147
**TOTAL**	96		181		277
**75+ Years**					
**Romantic Involvement/Desire**					
Married or cohabiting	24	57.04	6	11.82	30
Romantically involved	5	7.70	4	5.02	9
Unpartnered but desire romantic involvement	1	1.23	2	1.87	3
Neither has nor desire main romantic involvement	16	34.04	78	81.28	94
**TOTAL**	46		90		136

Table 2 also reveals important gender differences in romantic involvement patterns by age. Although the prevalence of marriage and cohabitation tends to rise and/or remain high among older African American men, marriage and cohabitation decreases precipitously with age for older African American women. For example, 54.0% of men aged 55–64, 61.9% of men aged 65–74, and 57.0% of men aged 75 and older were married/cohabiting. However, the opposite patterns were found for older African American women. Roughly 42.1% of those aged 55–64 were married/cohabiting, compared to 21.2% of those aged 65–74 and only 11.8% of those aged 75 and older. The prevalence of neither having nor desiring a main romantic involvement rises drastically across the later life course for older African American women. For instance, 31.4% of older African American women aged 55–64 neither had nor desired a main romantic involvement, a figure that increased dramatically for those aged 65–74 (58.6%) and those ages 75 and older (81.3%).

Table 3 presents multinomial logistic regression models of sociodemographic characteristics and health status on marriage and romantic involvement. Model 1 in Table 3 predicts the likelihood of having a main romantic involvement (vs. married/cohabiting). In this model, age, household income, and region are significantly associated with romantic involvement vs. marriage. Older respondents are less likely to be romantically involved and more likely to be married or cohabiting (OR = 0.96; 95% CI: 0.92, 1.00). Similarly, those with higher incomes are less likely to be romantically involved as opposed to married or cohabiting (OR = 0.91; 95% CI: 0.84, 0.98). Respondents who reside in the Northeast (OR = 2.38; 95% CI: 1.09, 5.23), North Central (OR = 3.02; 95% CI: 1.56, 5.85), and West (OR = 2.11; 95% CI: 1.10, 4.07) are more likely than Southerners to be romantically involved, whereas Southerners are more likely to be married.

**Table 3:** Weighted multinomial logistic regression analysis of demographic and self-rated health and romantic involvement/desire among Older African Americans, National Survey of American Life (N = 766).

	Romantically Involved Compared to Married/Cohabit	Desire Romantic Involvement Compared to Married/Cohabit	Neither Have Nor Desire Romantic Involvement Compared to Married/Cohabit
	Model 1	Model 2	Model 3
	RRR (95% C.I.)	RRR (95% C.I.)	RRR (95% C.I.)
Female (ref = male)	0.05 (0.00,7.43)	0.00 (0.00,0.77)*	0.05 (0.00,1.92)
Age	0.96 (0.92,1.00)*	0.88 (0.81,0.95)**	1.03 (0.99,1.07)
Age * Female	1.05 (0.97,1.14)	1.13 (1.02,1.26)*	1.07 (1.01,1.13)*
Household income	0.91 (0.85,0.98)*	0.87 (0.80,0.95)**	0.84 (0.77,0.92)***
Education	0.97 (0.88,1.06)	1.07 (0.93,1.23)	1.05 (0.94,1.16)
Region (ref = South)			
Northeast	2.38 (1.09,5.23)*	2.04 (0.53,7.88)	1.43 (0.74,2.76)
North Central	3.02 (1.56,5.85)**	3.45 (1.75,6.80)**	1.40 (0.66,2.98)
West	2.11 (1.10,4.07)*	1.01 (0.38,2.68)	0.90 (0.39,2.10)
Material Hardship	1.09 (0.83,1.45)	1.08 (0.84,1.39)	1.17 (0.91,1.50)
Self-Rated Health	1.18 (0.86,1.61)	1.06 (0.79,1.41)	0.90 (0.71,1.15)
F df = 30,4	7.91*		

RRR = Relative Risk Ratio, C.I. = Confidence Intervals, df = degrees of freedom

* *p* < 0.05;

** *p* < 0.01;

*** *p* < 0.001.

Model 2 in Table 3 predicts the likelihood of desiring a main romantic involvement (vs. married/cohabiting). Gender, age, the age x gender interaction, household income, and region are significantly associated with respondents’ desire for a romantic relationship compared to being married/cohabiting. The gender and age relationships must be interpreted in the context of the significant gender x age interaction. Across all age groups, men are more likely to be married/cohabiting, whereas women are more likely than men to be unmarried and desire to be in a romantic relationship. For both women and men, age is negatively associated with the odds of being married or cohabiting. However, the association is stronger for older African American women than men, indicating that as age advances, women’s odds of being married or cohabiting decreases faster than men’s odds of being married/cohabiting. This pattern is evident in the cross-tabular analysis (Table 2). (It is important to note that the number of people who are unmarried and desire to be in a romantic relationship is relatively small (n = 81) and extremely small at advanced ages, but this interaction is based on the differences in marriage/cohabitation by age and gender where the number of respondents is not an issue). Model 2 also reveals that respondents with higher household incomes are less likely to desire a romantic relationship and more likely to be married (OR = 0.87; 95% CI: 0.80, 0.95). In addition, residents of the North Central region are more likely to desire a romantic involvement than to be married, compared to respondents who reside in the South (OR = 3.45; 95% CI: 1.75, 6.80).

**Fig 1:**
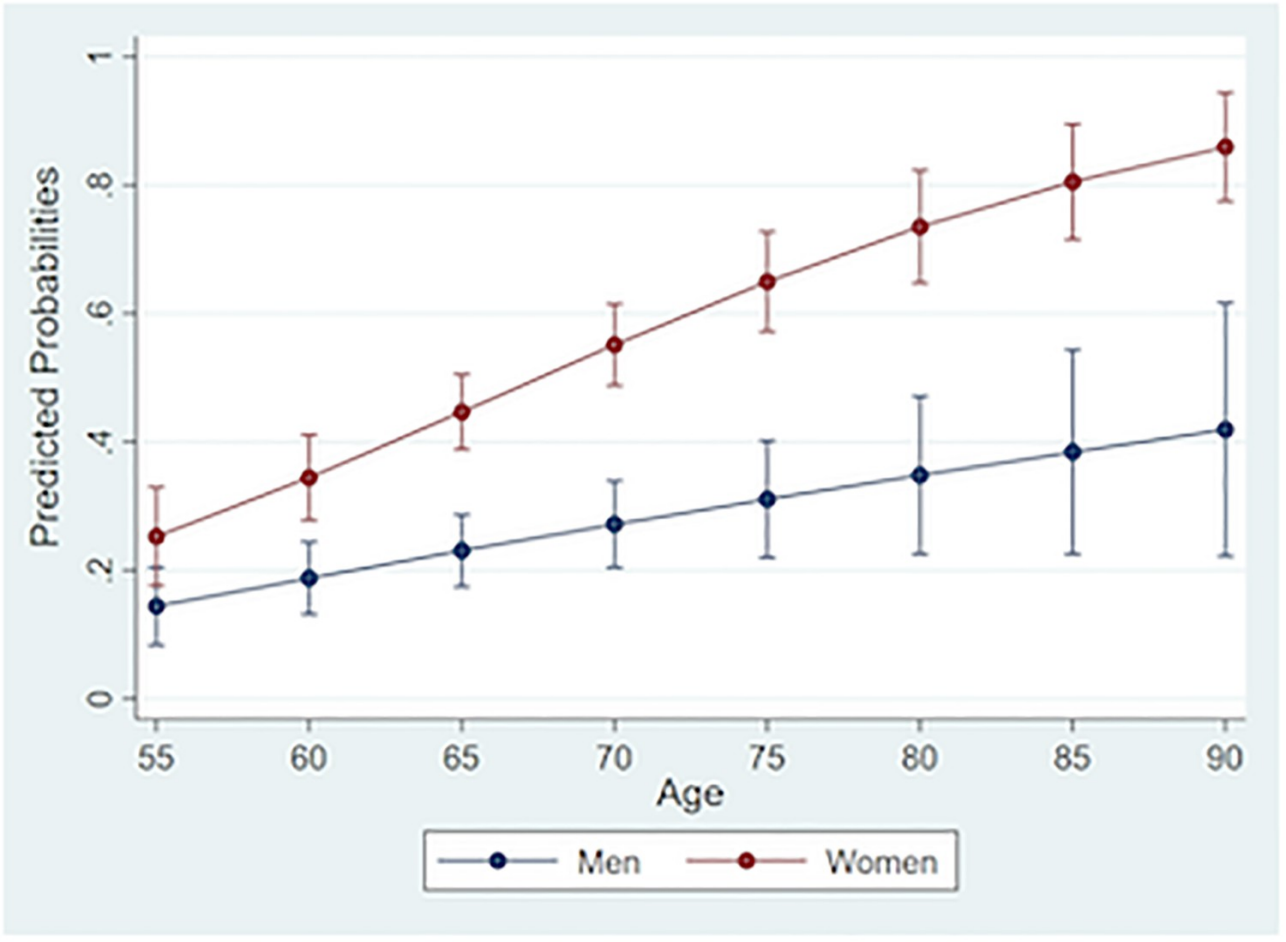
Predicted probabilities of neither having nor desiring a main romantic involvement vs. being married/cohabiting, by sex and age among older African Americans, National Survey of American Life (N = 766).

Model 3 in Table 3 presents the analysis comparing not having/desiring a romantic involvement with the likelihood of being married/cohabiting. The gender x age interaction is significant in this model (Fig 1) and shows three important findings. First, across age groups, African American women are more likely than their male counterparts to report that they neither have nor desire a romantic relationship. Second, older respondents are more likely than their younger counterparts to indicate that they do not have or desire a romantic relationship. However, this relationship is much stronger for African American women than men. This pattern is also evident in the cross-tabular analysis (Table 2) between age, gender and not having/desiring a romantic relationship. One out of four African American men age 75 and over indicate that they do not want a romantic involvement, in contrast to almost 9 out of 10 African American women aged 75 and over. Lastly, household income is negatively associated with neither having nor desiring a romantic relationship such that respondents with lower incomes are more likely to neither have nor desire a romantic relationship, whereas higher income respondents are more likely to be married (OR = 0.84; 95% CI: 0.77, 0.92; Model 3 in Table 3).

The analysis in Table 4 also presents the multinomial logistic regressions of sociodemographic factors and health on romantic involvement/desire. Unlike Table 3, instead of married/cohabiting, the comparison group is “neither have nor desire a main romantic involvement” (using married/cohabiting respondents as the comparison is redundant with Model 3 in Table 3). Two of the significant relationships in Table 4 involve age such that younger respondents are more likely to have a romantic involvement (OR = 0.93; 95% CI: 0.89, 0.97; Model 1) and more likely to report that they desire a romantic involvement (OR = 0.85; 95% CI: 0.78, 0.93; Model 2). Lastly, respondents in the North Central region are more likely to desire a romantic involvement than Southerners (OR = 2.46; 95% CI: 1.11, 5.45).

**Table 4:** Weighted multinomial logistic regression analysis of demographic and self-rated health and romantic involvement/desire among older African Americans, National Survey of American Life (N = 766).

	Romantically Involved Compared to Neither Has Nor Desires Romantic Involvement	Desire Romantic Involvement Compared to Neither Has Nor Desires Romantic Involvement
	Model 1	Model 2
	RRR (95% C.I.)	RRR (95% C.I.)
Female (ref = male)	0.95 (0.01,142.44)	0.02(0.00,12.64)
Age	0.93 (0.89,0.97)**	0.85 (0.78,0.93)***
Age * Female	0.99 (0.91,1.06)	1.06 (0.95,1.17)
Household income	1.08 (0.97,1.20)	1.03 (0.92,1.16)
Education	0.92 (0.83,1.03)	1.02 (0.90,1.16)
Region (ref = South)		
Northeast	1.67 (0.69,4.01)	1.42 (0.51,3.94)
North Central	2.15 (0.91,5.11)	2.46 (1.11,5.45)*
West	2.35 (0.64,8.65)	1.13 (0.28,4.47)
Material Hardship	0.94 (0.71,1.24)	0.92 (0.70,1.23)
Self-Rated Health	1.30 (0.98,1.73)	1.17 (0.89,1.53)
F df = 30,4	7.91*	

RRR = Relative Risk Ratio, C.I. = Confidence Intervals, df = degrees of freedom

* *p* < 0.05;

** *p* < 0.01;

*** *p* < 0.001.

## Discussion

This study investigated both the presence of romantic relationship status (whether marriage, cohabitation, or a main romantic involvement with a non-residential partner) and desire for romantic involvement among African Americans aged 55 and older. Roughly 1 in 10 older African American adults (11.3%) have a main (non-residential) romantic involvement, a group that is overlooked in governmental estimates that focus only on marital status and living arrangements among adults. Notably, this finding demonstrates that although older Blacks have relatively low level of marriage and cohabitation, a portion of those who are unmarried have romantic involvements. This finding is consistent with work in the 1980s, where 16.5% of older African Americans reported a romantic involvement [12].

Despite these findings, a large proportion of older African Americans (38.5%) are not connected to the marriage and dating markets. Among older African Americans who are not currently married or cohabiting, only 15.3% actively desire a romantic partnership, while the other 84.7% (representing 38.5% of the overall study population) indicate no desire for a main romantic involvement. Across all age groups, African American women are more likely than their male counterparts to report that they neither have nor desire a romantic relationship and this difference is particularly evident among the oldest African Americans. Although speculative, there are potential gender-specific reasons for this trend, which are consistent with sex-ratio disparity and social exchange theory.

As mentioned above, although the sex ratio begins decreasing universally for all race/ethnic groups, it becomes far more skewed at the later stages of the life course and drops most precipitously for African Americans. For example, between the ages of 65–69, the sex ratio dips to 76.7 Black men for every 100 Black women but by 80–84, it plummets to 52.7. At the oldest age group, 90 and older, there are only 30.9 Black men per 100 Black women [9]. Imbalanced sex ratios have substantial implications for gendered relationship dynamics, regardless of race. For example, in countries or subpopulations where the sex ratio is imbalanced, the sex that is in higher demand (i.e., the one with the fewest numbers relative to their opposite counterparts) yields greater influence in selecting a partner and establishing power dynamics within the relationship [39]. Under these circumstances, the group with fewer alternatives may have to “settle” for partners with less desirable characteristics while the group with more alternatives typically show less willingness to become and remain committed. Unpartnered older men benefit the most from increasingly imbalanced sex ratios (and therefore multiple options for dating and casual companionship) across the later life course. As such, they may choose to forego a main romantic involvement given their relatively greater options for romantic companionship.

The prevalence of neither having nor desiring a main romantic involvement greatly increased in later ages for older African American women. Consistent with social exchange theory, older African American women may perceive fewer benefits and more costs to initiating a romantic partnership with men who often experience worsening health earlier than they do. Indeed, caregiving for one’s partner—a responsibility disproportionately shouldered by women given men’s earlier health decline—is associated with a host of health burdens for women [40]. For older men, on the other hand, the marriage and dating markets include potential female partners who possess valued characteristics in terms of better health (i.e., life expectancy), as well as potential benefits associated with the provision of gendered domestic and caregiving duties. Within a social exchange framework, these valued characteristics of potential female partners might encourage men to initiate and maintain a romantic involvement.

The high rates of neither having nor desiring a romantic relationship among older unmarried African American woman is the result of several factors. First, older women may not want to enter a romantic relationship that involves providing care to an older man. Many older adults hold traditional gender role ideology [41,42], including the notion that women in married or dating relationships are primarily responsible for domestic responsibilities including cooking, cleaning, and laundry. This concern was expressed in a focus group on romantic involvement, with one older African American women stating, “Men want someone to wait on them, to be a servant, just take care of them” [12]. Consequently, one reason that many older African American women no longer desire to be in a romantic relationship is because the burden of domestic responsibilities outweighs the benefits of romantic involvement. This may particularly be the case for women who are widowed or who may have been in a long-term traditional relationship and are now no longer willing to accept that arrangement. Qualitative data from a study examining interest in remarriage among older widows in Oregon and California found that women typically cited a desire to avoid renewed domestic and caregiving responsibilities, often valuing their own freedom over any perceived benefits to remarriage [41].

Second, from a social exchange perspective, older women may be disinclined to have a romantic relationship with someone who is less attractive as a potential partner due to health issues. Although some older women may be willing to provide caregiving assistance for an ill romantic partner, for others, this potential burden may exclude them from consideration as a romantic partner. On the other hand, research indicates that older men who are ill are able to find a romantic partner [23]. In some instances, older women may be willing to partner with older men even if they are ill, which is consistent with sex-ratio imbalances in which older women outnumber eligible older men [23].

Third, older African American women are aware that the number of potential mates becomes increasingly smaller as they age. Consequently, stating that they do not desire a romantic partner may be a realistic acknowledgement of the lack of potential and desirable romantic partners in old age. Finally, regional differences indicated that Southerners were more likely to be married than either individuals who were romantically involved or who desired a romantic involvement. This is consistent with previous research findings that older African American southerners have a greater likelihood of being married due to higher numbers of African Americans and more favorable sex ratios in the south, coupled with a more traditional and religious milieu which is associated with marriage [12].

The decline in marriage over the past 50 years has important implications for older African Americans, especially older African American women. First, unmarried older African American women are much more likely to live alone [43] and they also have smaller informal social support networks than their married counterparts [44]. Consequently, they are at higher risk for placement in an assisted care facility because they do not have someone to help with some of the instrumental daily activities of life such as help with cooking, cleaning, paying bills and grocery shopping [45,46]. Second, unmarried older women have more than three times the poverty rate of married older women and older African American women have more than twice the poverty rate of older White women [47]. These patterns are due to a combination of lower income over the life course, coupled with a greater likelihood of living alone. The continued trend for decreasing marriage, coupled with increasing longevity, means that older African American women are at risk for experiencing higher rates of poverty.

Finally, it is important to acknowledge the general absence of research on marital and relationship behaviors and attitudes among older African American women and men. By-and-large, existing literature on African American marital and relationship attitudes and behaviors is primarily based on younger cohorts (i.e., college students, young and middle-aged adults). Topics such as attitudes and beliefs about marriage and intimate relationships, marital history and satisfaction, relationship quality, and partner characteristics and expectations in terms of education and income level, are just a few of the issues that are pertinent for furthering our understanding of marriage and romantic relationships among older African Americans.

This study has important strengths and limitations to consider. First, unlike most nationally representative datasets, the National Survey of American Life includes measures for both having a romantic relationship and, if unpartnered, the *desire for* romantic involvement. Although research on main romantic involvement (i.e., “living apart together”) has grown in recent years (e.g., [48,49]), most datasets used in family research include only measures for the presence or absence of marriage and other romantic relationships, not whether or not unpartnered respondents express an explicit desire for a romantic relationship. One exception is the American Changing Lives study, although their measure of desire was only administered to widowed individuals [19,50]. Additionally, only the NSAL has both a substantial number of older African Americans as well as these indicators of romantic relationships. With the NSAL, we were able to investigate a full range of demographic and health factors for a continuum of intimate partnerships and relationship statuses.

Second, most studies on romantic involvement include only a control variable for race, without explicit consideration of how the overall patterns vary for African Americans. As discussed earlier, the socio-ecological context of the dating and marriage markets for African Americans is quite distinct than that of Whites. It is therefore unwise to assume equivalent processes for African Americans and future research should continue to investigate potential unique patterns among this group. Indeed, even among the same Black racial category, there are significant differences in the correlates of relationship satisfaction, relationship longevity, and marital expectation between unmarried African Americans and Caribbean Blacks [35]. Although not explicitly focused on relationship desire, that study also found that age was inversely related to marriage expectations (i.e., the belief that the respondent will get married or remarried in the future) among African Americans, especially African American women [35]. Finally, we also add to a body of research focused on non-marital romantic relationships among Black Americans, especially given that marriage cannot be viewed as a panacea to eliminate social inequality faced by this group [51].

There are also two limitations to consider. First, NSAL data were collected between 2001–2003 and may not fully capture the full range of more contemporary romantic relationships. However, this paper represents the most recent update of an earlier study investigating the correlates of romantic involvement among a national sample of older African Americans [12]. Additionally, given the two strengths noted above (the inclusion of a measure for desire and the particular emphasis on African Americans), we argue that these findings are crucial to continue building the body of knowledge on African American families. Another limitation is that we use household income as opposed to personal income. This was done because of the high rate of non-response on the personal income question. Another limitation of this study was the inability to explore factors associated with relationship status and desires among older gay, lesbian, bisexual and other sexual and gender minorities, due to a lack of relevant information in the dataset. Future data collection efforts should prioritize sampling sexual minority populations.

### Conclusion

The deinstitutionalization of marriage has meant that marriage is no longer the defining social institution of American life [52]. This is increasingly the case for African Americans, and especially African American women. Moreover, being single in older age has social and health consequences. Among older adults who live alone, African Americans (33%) are twice as likely as Whites (16%) to live below the poverty line [53]. In addition to their greater risk of material deprivation, singlehood among older African Americans has important health implications, especially for women. For example, although Black men have lower life expectancy than Black women, Black women have higher rates of disability than White women and both Black and White men [54]. The increasing proportion of unmarried older African American women is at high risk for material deprivation and poorer health status. Given that spouses conventionally provide caregiving in older age, the increasing proportion of older African American women living outside of the context of marriage, cohabitation, and romantic relationships will require creative public policy solutions.
